# Shared Genetic Regulatory Networks Contribute to Neuropathic and Inflammatory Pain: Multi-Omics Systems Analysis

**DOI:** 10.3390/biom12101454

**Published:** 2022-10-11

**Authors:** Fang Ye, Li Du, Wenqi Huang, Sheng Wang

**Affiliations:** 1 Department of Anesthesiology, The First Affiliated Hospital, Sun Yat-Sen University, Guangzhou 510080, China; 2 Vitalant Research Institute, San Francisco, CA 94118, USA; 3 Department of Laboratory Medicine, University of California, San Francisco, CA 94143, USA; 4 Institute for Neurodegenerative Diseases, UCSF Weill Institute for Neurosciences, University of California, San Francisco, CA 94143, USA

**Keywords:** genetic regulatory networks, inflammatory pain, key drivers, biological pathways, multi-omics integration, neuropathic pain

## Abstract

The mechanisms of chronic pain are complex, and genetic factors play an essential role in the development of chronic pain. Neuropathic pain (NP) and inflammatory pain (IP) are two primary components of chronic pain. Previous studies have uncovered some common biological processes in NP and IP. However, the shared genetic mechanisms remained poorly studied. We utilized multi-omics systematic analyses to investigate the shared genetic mechanisms of NP and IP. First, by integrating several genome-wide association studies (GWASs) with multi-omics data, we revealed the significant overlap of the gene co-expression modules in NP and IP. Further, we uncovered the shared biological pathways, including the previously reported mitochondrial electron transport and ATP metabolism, and stressed the role of genetic factors in chronic pain with neurodegenerative diseases. Second, we identified 24 conservative key drivers (KDs) contributing to NP and IP, containing two well-established pain genes, *IL1B* and *OPRM1*, and some novel potential pain genes, such as *C5AR1* and *SERPINE1*. The subnetwork of those KDs highlighted the processes involving the immune system. Finally, gene expression analysis of the KDs in mouse models underlined two of the KDs, *SLC6A15* and *KCNQ5*, with unidirectional regulatory functions in NP and IP. Our study provides strong evidence to support the current understanding of the shared genetic regulatory networks underlying NP and IP and potentially benefit the future common therapeutic avenues for chronic pain.

## 1. Introduction

Neuropathic pain (NP) and inflammatory pain (IP) are two primary components of chronic pain, which is a common debilitating condition with a prevalence ranging from 30 to 50% in adults worldwide [1]. Poor chronic pain management has led to socioeconomic burdens and the opioid epidemic crisis [2,3]. NP is characterized by burning, tingling, electric shock-like pain with numbness or itching [4] and is mainly caused by damage to neurons involved in peripheral and central nervous system sensitization, including facial pain, trigeminal neuralgia, and multiple sclerosis pain [5]. In comparison, IP, a major clinical concern in diseases such as rheumatoid arthritis and osteoarthritis, is often accompanied by typical inflammatory symptoms, including redness, heat, swelling, and loss of function [6].

Previous studies have suggested pathways including ion channels, neurotransmitters, regulatory protein changes in neurons and glial cells involved in NP, and persistent neuroinflammatory or neuroimmune responses involved in IP [7,8]. Studies using multiple animal experiments have demonstrated common pathways, such as changes in cytokines/receptors, enhanced glutamate release and receptor function, disinhibition of the spinal dorsal horn, and glial cell activation in NP and IP [9]. Genetic studies have shown that genetic factors contribute to 16–50% of chronic pain [10,11,12], resulting in the identification of several pain genes, including *OPRM1*, *TRPV1*, *SCN9A*, *COMT*, *MTHFR*, *TNFA*, *GCH1*, *ESR1*, *ABCB1*, *P2RX7*, *CHRNA6*, and *CACNG2* [13,14,15]. However, the shared genetic mechanism underlying NP and IP has never been studied.

To date, various pharmacological strategies have been developed to treat these two types of chronic pain. Typically, antidepressants and antiepileptic drugs are effective for NP [16], while non-steroidal anti-inflammatory drugs are more commonly used for IP [17]. Although some current drugs have cross-analgesic effects, the underlying mechanism is still unclear. Moreover, effective drug treatments against both NP and IP with few side effects are still lacking [18].

Here we integrated genetic signals from recent genome-wide association studies (GWASs) of NP and IP and multi-omics data to investigate the shared genetic mechanisms in NP and IP. Thus, our findings may provide system-level insights into NP and IP from a genetic perspective and potential therapeutic and preventive targets for chronic pain.

## 2. Materials and Methods

### 2.1. Data Collection and Filtration of GWASs on NP and IP

We obtained the summary-level data from 21 public GWASs [19,20]. Chronic pain-related phenotypes were selected using the keyword “chronic pain”. Each pain phenotype was classified by the definition of NP and IP by ICD11 and previous literature [4,5,6,7,8]. In detail, the traits with trigeminal neuralgia, sciatica, neuroglia and neuritis, multiple sclerosis, and neuropathic facial pain were classified as NP; traits with rheumatoid arthritis, osteoarthritis, and ankylosing spondylitis were classified as IP. Only traits with a sample size > 10,000 and case count > 500 were selected. We only included the samples with European ancestry due to the data availability. The details are listed in Table S1. All the GWAS results in VCF format were processed with in-house scripts (https://github.com/swang05/chronicpain.git, accessed on 25 February 2022) to (1) include minor allele frequency > 0.05 and imputation quality > 0.3 and (2) rank all the SNPs by the −log10 *p*-values in each study to select the top 50% most significant loci [21]. We further applied a linkage disequilibrium (LD) filter to only keep one SNP in a strong LD block. Specifically, we calculated the LD r-square (r^2^) for each pair of adjacent SNPs by plink 1.9 [22] with data of 503 European samples from the 1000 Genomes Phase 3 European data as reference [23]. For each SNP pair, we pruned one if the estimated r^2^ > 0.5 to only keep the most significant signal based on the associated *p* values. The LD filtration step was repeated until no more SNPs were excluded in an iteration.

### 2.2. Curation of Association between SNPs and Genes

To understand the functions of the GWAS SNPs, we annotated the filtered SNPs with varied supportive datasets. We obtained the curated cis-eQTL data from Genotype-Tissue Expression (GTEx) project release v8 [24,25] for 49 different tissues. This dataset contains well-annotated SNP–gene association based on permutations. Only associations with qval ≤ 0.05 were retained in the analysis. Additionally, we attempted to link the SNPs to the nearby genes. We first defined the association between the SNP and the genes within 50 kb and kept the SNP–gene associations with sufficient evidence (score must be greater than 4, which indicates the supportive evidence from both transcriptional factor binding and DNase peak) in the Regulome database [26].

### 2.3. Construction of Gene Co-Expression Modules

We obtained the gene expression data from GTEx [24,25] for 15 tissues that are potentially associated with NP and/or IP, namely brain amygdala, brain hippocampus, brain hypothalamus, brain cortex, brain frontal cortex, brain anterior cingulate cortex, brain caudate, brain cerebellar hemisphere, brain cerebellum, brain nucleus accumbens, brain putamen, brain substantia nigra, brain spinal cord, tibial nerve, and whole blood. After checking the potential batch effects introduced by age and sex, we constructed co-expression modules by weighted gene co-expression network analysis (WGCNA) [27] using samples from 15 tissues, each of which includes more than 80 donors that are supposed to be healthy population control [28,29], in R (version 4.1.0) with WGCNA package (version 1.70-3) (Figures S1–S15). We excluded the modules with less than 10 genes in the downstream analysis. As a result, we generated a total of 799 co-expression modules. The biological processes of each module were annotated with 1615 canonical Reactome pathways and 186 canonical Kyoto Encyclopedia of Genes and Genomes (KEGG) pathways from MSigDB [30,31]. Statistical significance was determined by Fisher’s exact test with Bonferroni-corrected *p* < 0.05.

### 2.4. Identification of Associated Modules in NP and IP

We conducted marker set enrichment analysis (MSEA, Mergeomics R package, version 1.22.0, Open source, Los Angeles, CA, USA) to identify overrepresented modules in NP and IP for each GWAS [21]. Three inputs are required for MSEA: (1) filtered SNPs, (2) SNP–gene mapping information, and (3) co-expression modules. Briefly, MSEA evaluated the enrichment of the disease SNPs in each co-expression module compared with random status with chi-square statistics. The Benjamini–Hochberg FDRs were calculated across all tested modules for each GWAS.

We further employed meta-MSEA analysis to meta-analyze the signals across different studies to obtain robust signals. Meta-MSEA will calculate the meta-Z-score by Stouffer’s Z score method utilizing the estimated *p* values from MSEA and then convert it back to the meta-*p*-value. The Benjamini–Hochberg method was applied to generate the final meta-FDR. We only considered the co-expressed modules with meta-FDR < 0.05 for the downstream analysis [32]. 

### 2.5. Evaluation of Shared Genetic Mechanism between NP and IP

We assessed the genetic overlap between NP and IP utilizing significant co-expression modules identified with meta-MSEA. First, we compared the significant modules associated with NP and IP and identified the shared ones in both conditions. Next, for the modules specific in NP and IP, we took advantage of the annotations of the modules to investigate whether these condition-specific modules indeed overlapped in biological pathways. The enrichments of the overlapping modules were estimated through permutation tests by randomly assigning the significant modules. We defined a “success” as the overlap for the permuted lists being no less than the observation. The *p* values were estimated by the number of “successes” in 100,000 permutations. The enrichment fold was obtained with the observation divided by the median of the permutations.

To identify the core genes contributing to both conditions, we constructed supernets based on the pathway analysis. We aggregated the modules for each pathway in NP and IP separately. We then merged the modules from NP and IP for an overlapping pathway where more than 15% of genes are overlapped in both NP and IP and created 47 functionally categorized supernets [33].

### 2.6. Identification and Consolidation of Key Drivers in NP and IP

The co-expression modules identified by meta-MSEA associated with NP and/or IP and mapped to graph gene–gene interactions in 12 NP- and/or IP-related tissues were obtained from the GIANT project [34]. We performed the weighted key driver analysis with the wKDA function implemented in the Mergeomics package. Briefly, wKDA will first identify potential hub genes with sufficient numbers of genes connected, followed by building subnetworks with those hub genes with one-edge neighbors. All the genes of each supernet defined above will be mapped to the subnetworks to evaluate whether these genes are overrepresented in a certain subnetwork. The enrichment of each supernet was estimated through permutation tests, and *p* values were generated based on the null distribution. We identified the key drivers (KDs) based on the FDR < 0.05 and ranked the KDs by the number of tissues with each KD. We primarily focused on the KDs with supportive evidence in at least two tissues, namely conservative KDs.

### 2.7. Systematic Analyses of Conservative KDs

We generated a subnetwork with the conservative KDs with the one-edge neighbors using the protein interaction information from PCNet [35]. PCNet has integrated 21 different network databases and is carefully curated to increase the confidence of the interactions. We constructed the network for the KDs with the interactions in PCNet and visualized the network using Cytoscape v 3.3.0 (Open source, San Diego, CA, USA) [36]. For the nodes, we only kept the ones with functional SNPs achieving genome-wide significance (*p* < 5 × 10^−8^) [37]. The subnetwork was annotated with Reactome and KEGG with a Fisher’s exact test with Bonferroni-corrected *p* < 0.05. Gene ontology (GO) analysis on the KDs was performed using the GO Database [38,39].

### 2.8. Changes in KD Expression in NP and IP Mouse Models 

We also determined the gene expression changes in two mouse models: spared nerve injury (SNI) for NP and complete Freund’s adjuvant (CFA) model for IP. We obtained the gene expression data from public data [40] for the KDs and calculated the enrichment factor by comparing the gene expression level in the mouse model versus the controls (CTR) after normalization by expression in blood:

$$\mathrm{EnrichFactor}\;=\frac{\mathrm{Expression}([\mathrm{SNI}|\mathrm{CFA}]\_\mathrm{tissue})/\mathrm{Expression}([\mathrm{SNI}|\mathrm{CFA}]\_\mathrm{blood})}{\mathrm{Expression}\left(\mathrm{CTR}\_\mathrm{tissue}\right)/\mathrm{Expression}\left(\mathrm{CTR}\_\mathrm{blood}\right)}$$

where tissue could be the whole brain, dorsal root ganglia, or spinal cord.

## 3. Results

### 3.1. NP- and IP-Related Co-Expression Modules Are Significantly Overlapped

**Figure 1:**
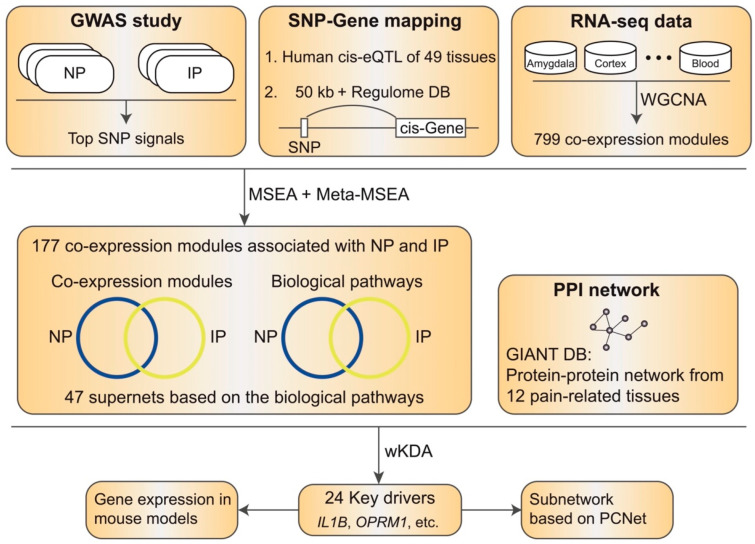
Study schema. We integrated the genetics and functional genomics datasets to identify NP- and IP-associated co-expression modules. Briefly, GWASs of NP and IP were obtained from public databases. A comprehensive list of tissue-specific functional genomic datasets was compiled, including 799 co-expressed modules and SNP-to-gene association. MSEA and meta-MSEA were performed to identify significantly overrepresented modules. The significant modules were annotated to reveal the common pathways of NP and IP. Using the modules, we constructed 47 supernets based on the shared biological processes. We then carried out wKDA to identify the conservative key drivers by integrating protein interaction networks from multiple pain-related tissues. The final KDs were mapped to PCNet to build a one-edge subnetwork and checked with the gene expression regulations in mouse models of neuropathic and inflammatory pain.

**Figure 2:**
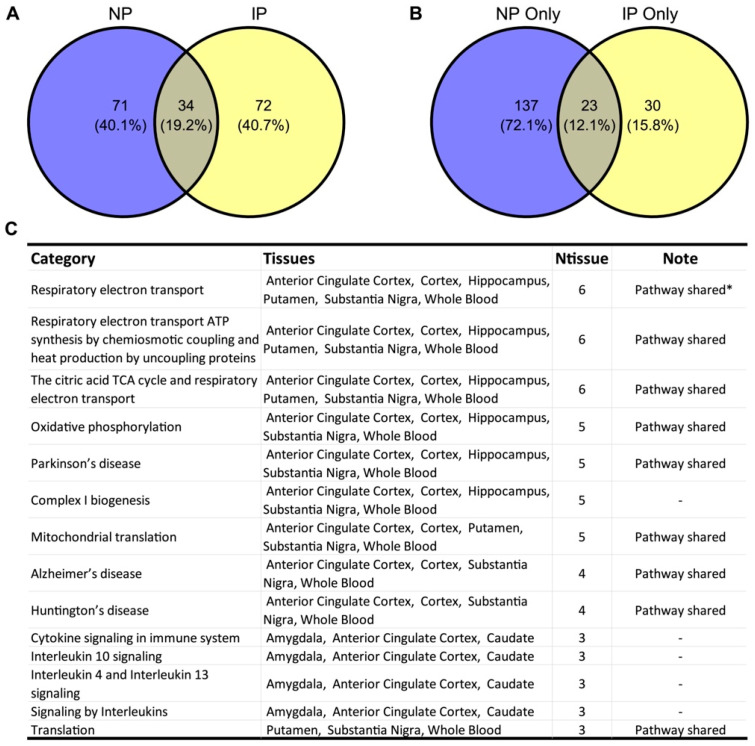
NP- and IP-associated co-expression modules and biological pathways are highly overlapped. (**A**) Count of significant modules in NP and IP; (**B**) count of functional categories from NP-only and IP-only modules; (**C**) overlap of top functional categories by NP and IP modules. Only pathways that appeared in at least three tissues are shown. Ntissue: number of tissues with the category; * pathway shared: pathways were also indicated in condition-specific modules in both NP and IP, i.e., the overlapped categories in (**B**).

We obtained RNA-seq data from GTEx v8 for 15 NP- and IP-associated tissues [24,25]. Utilizing weighted gene co-expression network analysis (WGCNA), we defined 799 co-expression modules with more than 10 genes from 15 NP- and/or IP-associated tissues (Figure 1, see Section 2). To identify the modules that are highly correlated with the two conditions, we integrated the SNPs from recent GWASs for NP and IP specifically [41] to perform the marker set enrichment analysis (MSEA) [21]. Briefly, we extracted the top SNPs from each GWAS and mapped them to the corresponding genes with known eQTL or genomic coordinate information together with evidence from Regulome. To reduce the bias introduced by multiple SNPs in a linkage block targeting the same gene, we further pruned adjacent SNPs based on the LD with public data [42]. MSEA was subsequently carried out in each tissue to evaluate the enrichment of the potential disease SNPs in each module with a chi-square-like statistic. After meta-analyzing the outputs across all the tissues, we identified 105 and 106 modules significantly associated with NP and IP, respectively (false discovery rate (FDR) < 0.05). Expectedly, we observed 34 modules in both conditions, which is statistically significant through the permutation test (*p* < 1 × 10^−5^, enrichment fold 2.62, Figure 2A), though there are still several modules that seem to be condition-specific. 

### 3.2. Shared Biological Pathways Contribute to NP and IP

We annotated the co-expression modules with the canonical pathways derived from Reactome and KEGG databases [30,31]. The shared modules that are associated in at least three different tissues underlined several pathways involving ATP metabolism, such as respiratory electron transport, ATP synthesis, the citric acid TCA cycle, oxidative phosphorylation, and complex I biogenesis (Figure 2C, Table S2). Surprisingly, we noticed several neurodegenerative diseases were also showing up, including Parkinson’s disease, Alzheimer’s disease, and Huntington’s disease.

We further explored the pathways implicated by the condition-specific modules. Of the 190 significant biological processes of those modules, 23 were shared in both conditions (14% in NP and 43% in IP, Figure 2B), most of which overlapped with the annotations using the shared modules, including mitochondrial electron transport, ATP metabolism, and neurodegenerative diseases (Figure 2C).

### 3.3. wKDA Identified 24 Conservative KDs Shared in NP and IP

We next identified the key drivers (KDs) contributing to NP and IP. Since the co-expression modules define the functional correlations exclusively, we involved the topological information from the GIANT protein networks of 12 NP- and/or IP-relevant tissues [34]. The condition-specific modules still shared a certain level of biological process, and therefore, instead of using shared modules only, we constructed 47 supernets with the modules that are with the same biological processes in NP and IP. With weighted key driver analysis (wKDA) implemented in Mergeomics [21], we firstly identified tissue-specific KDs that are consistently captured in both NP and IP with Bonferroni-corrected *p*-value < 0.05. The conservative KDs were subsequently defined as KDs detected in at least two tissues, yielding 24 conservative KDs (Figure 1 and Table 1). Notably, two KDs, SERPINE1 and ILB1, were captured in more than five pain-related tissues, suggesting these two KDs to be closely involved in pathogenesis [43]. 

Additionally, we further compared the top KDs with known pain genes obtained from the Pain Gene Resource of the International Association for the Study of Pain (IASP) Pain Research Forum [13], which included 94 pain-associated genes and phenotypes. There are two conservative KDs (IL1B and OPRM1) that were reported as pain genes previously (permutation test *p* = 0.0047, enrichment fold = 19.67). The significant overlap with known pain genes further strengthens our confidence that the identified conservative KDs are involved in pain pathogenesis.

**Figure 3:**
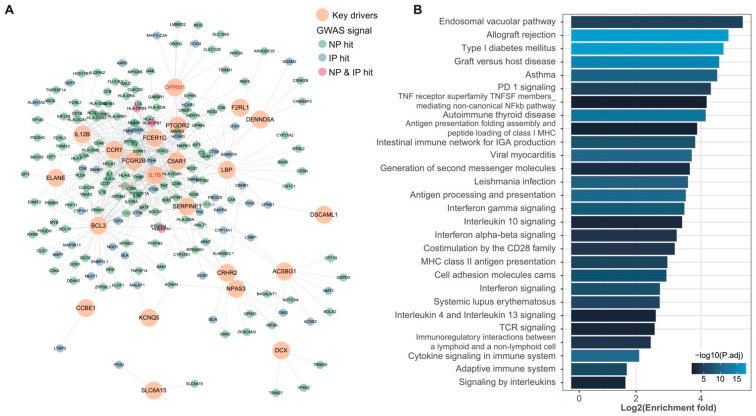
Key drivers and subnetworks for the NP/IP-associated modules. (**A**) Top KD subnetworks with GWAS hits (*p* < 5 × 10^−8^ as reported in GWAS Catalog). KD: key driver; known pain-related genes are indicated in the red font. (**B**) Pathway analysis of the genes involved in the subnetwork in (**A**).

Further, we generated an integrated network by involving the conservative KDs and their one-edge neighbors according to the more conservative interaction information from PCNet, which was carefully curated after integrating 21 network databases [35]. We found the KDs showed a direct connection to 141 genome-widely significant GWAS hits in NP (21% of total GWAS hits) and 47 in IP (13% of total GWAS hits) (Figure 3A). Pathway analysis of the genes involved in this network highlighted the processes related to the immune system (Figure 3B). GO analysis presented significant enrichments on the neuron projection membrane (GO: 0032589, enrichment fold = 28.02, Bonferroni’s corrected *p* = 0.0026) and neuronal cell body (GO:0043025, enrichment fold = 4.18, Bonferroni’s corrected *p* = 0.015).

### 3.4. Shared KDs Significantly Overlapped with Known Pain Genes

**Figure 4:**
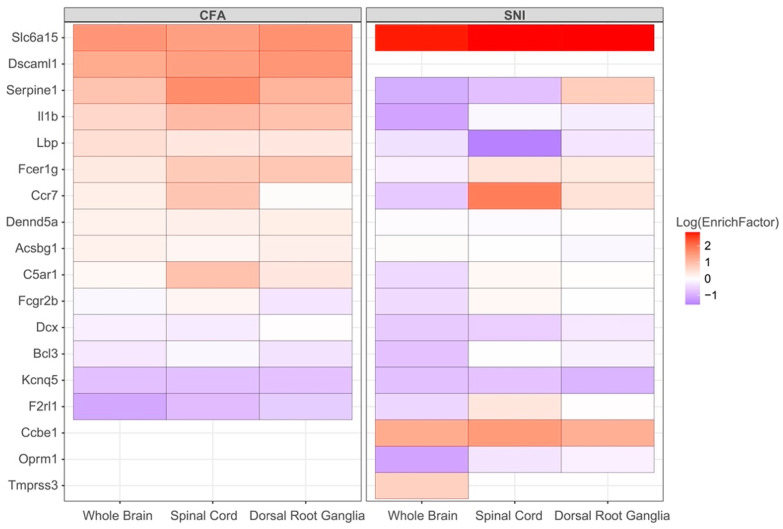
Differential gene expression in mouse models. Top: pain models, Bottom: tissue, CFA: complete Freund’s adjuvant, SNI: spared nerve injury. EnrichFactor: enrichment factor, shown as log-scaled values.

We identified the changes in KD expression in the pain-related tissue of NP and IP mice models, and various gene expression patterns of KDs were observed between NP and IP. In detail, a classic pain gene, IL1B, was generally upregulated in the IP model while downregulated in the NP model. Moreover, in IP mice, SERPINE1 was upregulated in the brain and spinal cord while downregulated in NP mice. In contrast, C5AR1 was upregulated in the spinal cord of IP animals but did not change in the spinal cord of NP animals. Remarkably, SLC6A15 and KCNQ5 exhibited unidirectional expression patterns, with upregulation and downregulation, respectively (Figure 4).

## 4. Discussion

Accumulating multiple-dimensional evidence to identify the shared genetic risks underlying relevant diseases has proven to be a promising way forward to elucidate disease networks and predict therapeutics [21,44]. For this reason, we integrated genetic signals and multi-omics data to investigate the shared genetic mechanisms in NP and IP. Our results highlighted the role of several common pathways in NP and IP. Moreover, we identified the top KDs strongly associated with NP and IP in multiple pain-related tissues. We further analyzed the KDs’ expression in mouse models of NP and IP and determined two of the KDs with unidirectional regulatory functions in NP and IP. 

Firstly, we identified several biological pathways significantly overrepresented in both NP and IP, including the previously reported mitochondrial electron transport [45] and ATP metabolism [46]. Moreover, we revealed three neurodegenerative diseases, Parkinson’s disease, Alzheimer’s disease, and Huntington’s disease, as the shared co-expression modules in NP and IP, positing the connection between neurodegenerative diseases and chronic pain. It is generally known that chronic pain is common in the population with neurodegenerative diseases, with a prevalence of 40–60% [47,48,49]. The mechanisms of chronic pain in neurodegenerative diseases have been extensively investigated [50,51,52,53]. Genetic factors such as *SCN1B-SCN4B* and *COMT* have been reported to contribute to chronic pain in neurodegenerative disease [54,55,56]. Conversely, previous integrated analysis of GWAS data found no genetic association between chronic pain and neurodegenerative disease [57]. Our study supports the role of the genetic factor for chronic pain in neurodegenerative disease. The specific mechanism remains to be further studied.

Secondly, combining the protein interaction data, we identified 24 conservative KDs shared by NP and IP in multiple tissues. Recent studies have found that genetic factors in pain susceptibility [58] and shared across chronic pain conditions [59] act through mechanisms within the brain. Remarkably, we revealed that the KDs shared by NP and IP are mainly expressed in the amygdala, caudate nucleus, caudate putamen, cerebellum, frontal lobe, hippocampus, substantia nigra, and other brain tissues (Table 1), which echoes the previous study. Notably, the identified KDs contain two well-established pain genes, *IL1B* [60,61] and *OPRM1* [62,63,64], adding confidence to our analyzed results. Moreover, we identified another interleukin family member, *IL12B*, as a KD, emphasizing the involvement of interleukins in NP and IP pathology. Moreover, the directly connected subnetwork of these KDs enriching substantial GWAS signals highlighted the role of regulation in the immune system in NP and IP. A recent study has revealed dual neuronal and immunological etiology for pain susceptibility using GWAS meta-analysis [58]. Interestingly, although another study reported no association between chronic pain and inflammatory cytokines [59], substantial evidence has linked inflammation and the immune system to multiple chronic pain conditions [65,66]. Consistent with Evelina Mocci et al.’s analysis [58], we further highlight the role of regulation in the immune system and neuronal function shared by NP and IP. For instance, it has been reported that the complement system significantly contributes to the development of NP and IP, but the underlying mechanisms are poorly investigated [67,68,69]. We found that *C5AR1* is a shared KD of NP and IP. This finding is supported by previous research showing that complement component 5a (C5a) induces mechanical hypersensitivity by activating a macrophage–neuron signaling cascade involving TRPV1 and CGRP receptors [70]. Accordingly, the C5a receptor 1 antagonists PMX53 and PMX205 can be further investigated as new targets for regulating NP and IP [71,72]. Somewhat unexpectedly, asthma and diabetes were revealed in our pathway analysis of the KDs’ subnetwork, which was confirmed by Evelina Mocci et al.’s analysis [58]. Moreover, these two un-pain traits were further demonstrated to increase pain susceptibility.

Additionally, our study reveals that *SERPINE1* is a shared key gene of NP and IP. In recent years, an increasing number of studies have focused on the function of plasminogen activator inhibitor-1 (PAI-1), encoded by *SERPINE1*, in neurological diseases, independent of its role as a tissue-type plasminogen activator [73,74,75]. However, few studies reported the role of *SERPINE1* in chronic pain. Our study highlights the functional role of *SERPINE1* in chronic pain from a genetic perspective, which we speculate may be caused by *SERPINE1* mutation leading to aberrant neurotransmitter metabolism and altered synaptic plasticity [73,75,76].

Thirdly, gene expression analysis of the KDs in mouse models shows varied changes in the pain-related tissues under NP and IP development. *SERPINE1* was upregulated in the brain and spinal cord in IP mice while downregulated in NP mice. In contrast, *C5AR1* was upregulated in the spinal cord of IP animals but did not change in the spinal cord of NP animals. However, we found that two genes, *SLC6A15* and *KCNQ5*, exhibit unidirectional regulation in the mouse models of NP and IP, respectively. *SLC6A15* encodes neuronal amino acid transport and was previously reported as a depression gene [77]. In vitro experiments demonstrated that deficiency of *SLC6A15* reduced the levels of proline and other neutral amino acids in hippocampus neurons, whereas *SLC6A15* overexpression increased intraneuronal glutamate concentrations [78], suggesting that *SLC6A15* positively regulates neuronal excitability. In addition, the neurites in *SLC6A15* knockout neurons grow faster with improved mitochondrial function, suggesting that *SLC6A15* negatively regulates nerve growth [79], thereby promoting the initiation and development of neuropathic pain. Our analysis of animal data showed that the expression of *SLC6A15* was upregulated in the brain, spinal cord, and DRG in IP and NP mice, which corroborated the above results.

In comparison, *KCNQ5*, which encodes the KCNQ channel subtype Kv7.5, is widely distributed in the nervous system and regulates resting potential and nociceptor excitability [80]. The opening of Kv7.5 on the presynaptic membrane helps maintain the negative membrane potential and reduces neurotransmitter release [81]. The potent Kv7.5 stimulator gabapentin is a classic drug for neuropathic pain treatment [82]. Another Kv7.5 activator, retigabine, can relieve CFA-induced IP in the mice model [83]. Our results from animal models show that the expression of *KCNQ5* is downregulated in the nervous tissue of both IP and NP animals, which is consistent with previous findings. In humans, missense mutations in *KCNQ3*, another member of the KCNQ family, have been reported to help with pain relief in patients with hereditary erythematosus [84]. However, the function of *KCNQ5* in chronic pain has not yet been elucidated. Our integrated analysis validates *KCNQ5* as a key driver in regulating NP and IP. Taken together, the unidirectional changes of the gene expression implicated in *SLC6A15* and *KCNQ5* may be tractable common therapeutic targets for chronic pain. 

Although our work greatly extends the understanding of the shared mechanism between NP and IP, several limitations should be mentioned. First, since most of the GWASs on chronic pain were carried out with populations of European ancestry, whether our observation is sensitive to population stratification is still undetermined. Following studies with non-European samples are critical to verify our results. Second, though we include various types of NP, such as facial pain and sciatica, the lack of multiple types of IP still enervates our conclusions; i.e., we are uncertain whether the common pathways and KDs revealed can be extrapolated to all types of IP. In addition, as specific information on the clinical severity score was lacking, we did not incorporate clinical severity scores into our analyses. Therefore, our data only demonstrated that these genes are involved in the NP and IP but cannot explain their contribution to the severity of pain. Last but not least, due to a lack of suitable samples, we did not identify the effect of specific cell types on changes in KDs’ expression, which should be taken into account in future prospective work in the field. Nonetheless, our work provides a systems-level understanding of the two main components of chronic pain. It potentially provides therapeutic and predictive targets to help achieve system-wide relief of chronic pain.

## 5. Conclusions

In summary, by integrating several genome-wide association studies (GWASs) with multi-omics data, we revealed the significant overlap of the gene co-expression modules in NP and IP. Further, we uncovered several shared biological pathways, including the previously reported mitochondrial electron transport and ATP metabolism, and stressed the genetic role in chronic pain in neurodegenerative disease. Second, we identified 24 vital conservative drivers (KDs) contributing to NP and IP, including two well-established pain genes, *IL1B* and *OPRM1*, and some novel potential pain genes, such as *C5AR1* and *SERPINE1*. The subnetwork of those KDs highlighted the processes involving the immune system. Finally, gene expression analysis of the KDs in mouse models underlined two of the KDs, *SLC6A15*, and *KCNQ5*, with unidirectional expression in NP and IP, which serve as potential targets for future medicine design for chronic pain. Altogether, our study provides strong evidence to support the current understanding of the shared genetic regulatory networks underlying NP and IP and potentially benefit the future design of the common therapeutic avenues for chronic pain.

## Figures and Tables

**Table 1:** Conservative KDs identified in NP and IP.

Gene	Tissue	Ntissue	If Known
*SERPINE1*	amygdala, blood, caudate nucleus, caudate putamen, cerebellum, frontal lobe, hippocampus, spinal cord, substantia nigra	9	no
*IL1B*	blood, caudate nucleus, caudate putamen, cerebellum, frontal lobe, hippocampus, spinal cord, substantia nigra	8	yes
*C5AR1*	amygdala, caudate nucleus, hippocampus, substantia nigra	4	no
*CCR7*	amygdala, cerebellar cortex, frontal lobe, hippocampus	4	no
*ACSBG1*	amygdala, caudate nucleus, substantia nigra	3	no
*BCL3*	blood, frontal lobe, nucleus accumbens	3	no
*DCX*	caudate nucleus, frontal lobe, substantia nigra	3	no
*ELANE*	caudate putamen, hippocampus, nucleus accumbens	3	no
*FCER1G*	caudate nucleus, hypothalamus, spinal cord	3	no
*IL12B*	caudate putamen, frontal lobe, hypothalamus	3	no
*OPRM1*	caudate putamen, hypothalamus, nucleus accumbens	3	yes
*SLC6A15*	caudate putamen, hypothalamus, nucleus accumbens	3	no
*CCBE1*	amygdala, caudate nucleus	2	no
*CRHR2*	hypothalamus, nucleus accumbens	2	no
*DENND5A*	amygdala, spinal cord	2	no
*DSCAML1*	cerebellar cortex, substantia nigra	2	no
*F2RL1*	blood, caudate nucleus	2	no
*FCGR2B*	amygdala, cerebellum	2	no
*KCNQ5*	amygdala, caudate putamen	2	no
*LBP*	cerebellum, hippocampus	2	no
*NPAS3*	frontal lobe, substantia nigra	2	no
*PTGDR2*	caudate putamen, hypothalamus	2	no
*TMPRSS3*	caudate putamen, substantia nigra	2	no
*TRPC7*	nucleus accumbens, substantia nigra	2	no

Ntissue: number of tissues; If Known: whether gene is a known pain gene.

## Data Availability

GWAS datasets: https://gwas.mrcieu.ac.uk (accessed on 19 July 2021); GTEx project: https://www.gtexportal.org; 1000 Genomes project: https://www.internationalgenome.org; RegulomeDB: https://www.regulomedb.org; GIANT database: https://giant.princeton.edu; mouse model datasets: https://journals.lww.com/pain/Abstract/2019/04000/Genetic_pathway_analysis_reveals_a_major_role_for.17.aspx.

## Supplementary Materials

The following supporting information can be downloaded at: https://www.mdpi.com/article/10.3390/biom12101454/s1, Figures S1–S15: Cluster dendrogram of different tissues. Figure S1: Brain amygdala; Figure S2: Brain hippocampus; Figure S3: Brain hypothalamus; Figure S4: Brain cortex; Figure S5: Brain Frontal cortex; Figure S6: Brain Anterior cingulate cortex; Figure S7: Brain caudate; Figure S8: Brain cerebellar hemisphere; Figure S9: Brain cerebellum; Figure S10: Brain nucleus accumbens; Figure S11: Brain putamen; Figure S12: Brain substantia nigra; Figure S13: Brain spinal cord; Figure S14: Nerve tibial; Figure S15: Whole blood. Figure S16: *C5ar1* and *Serpine1* mRNA were downregulated in the ipsilateral spinal cord after SNI. Table S1: Descriptive characteristics of the study cohorts; Table S2: Overlap of independent functional categories by NP and IP modules.
