# Supplementary material for: Shared Genetic Regulatory Networks Contribute to Neuropathic and Inflammatory Pain: Multi-Omics Systems Analysis
Source: Biomolecules. 2022 Oct 11;12(10):1454. doi: 10.3390/biom12101454 (PMC9599593; doi:10.3390/biom12101454)
Supplement: Supplementary file 1 [file biomolecules-12-01454-s001.zip › biomolecules-1926396-supplementaryS1.PDF]

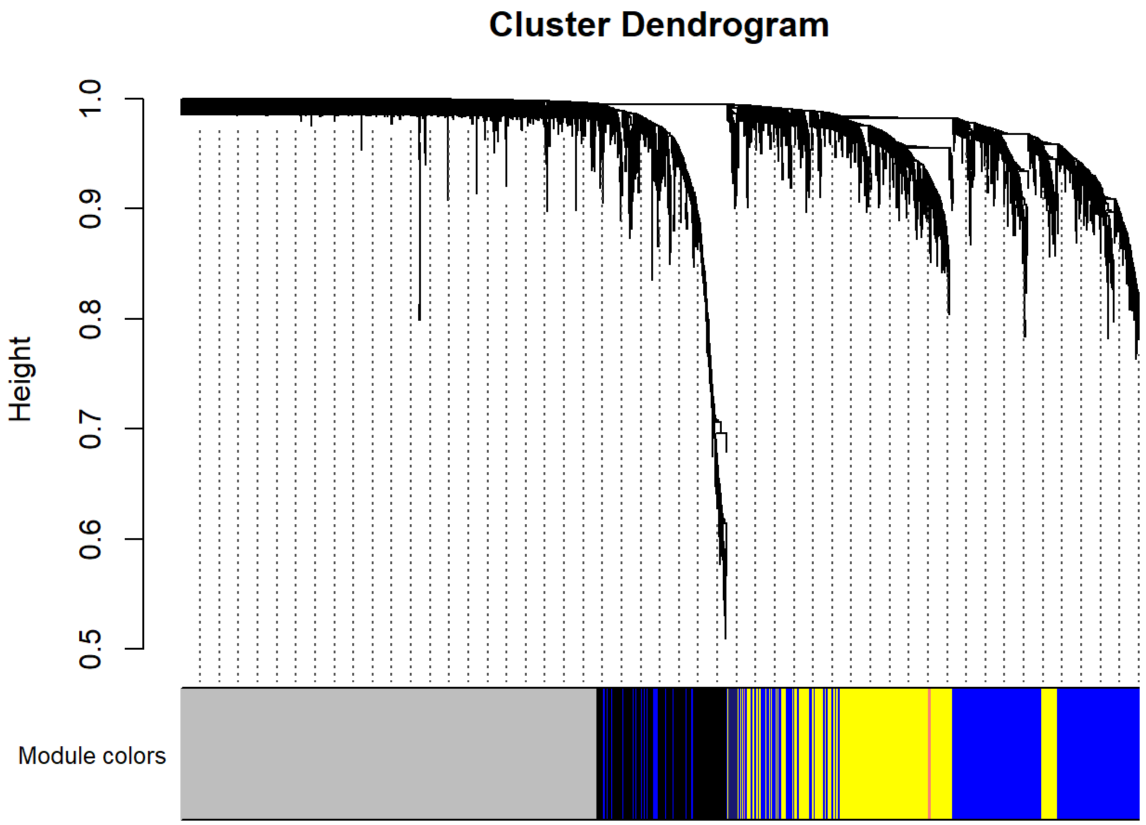

Supplementary Figure S1. Brain amygdala.

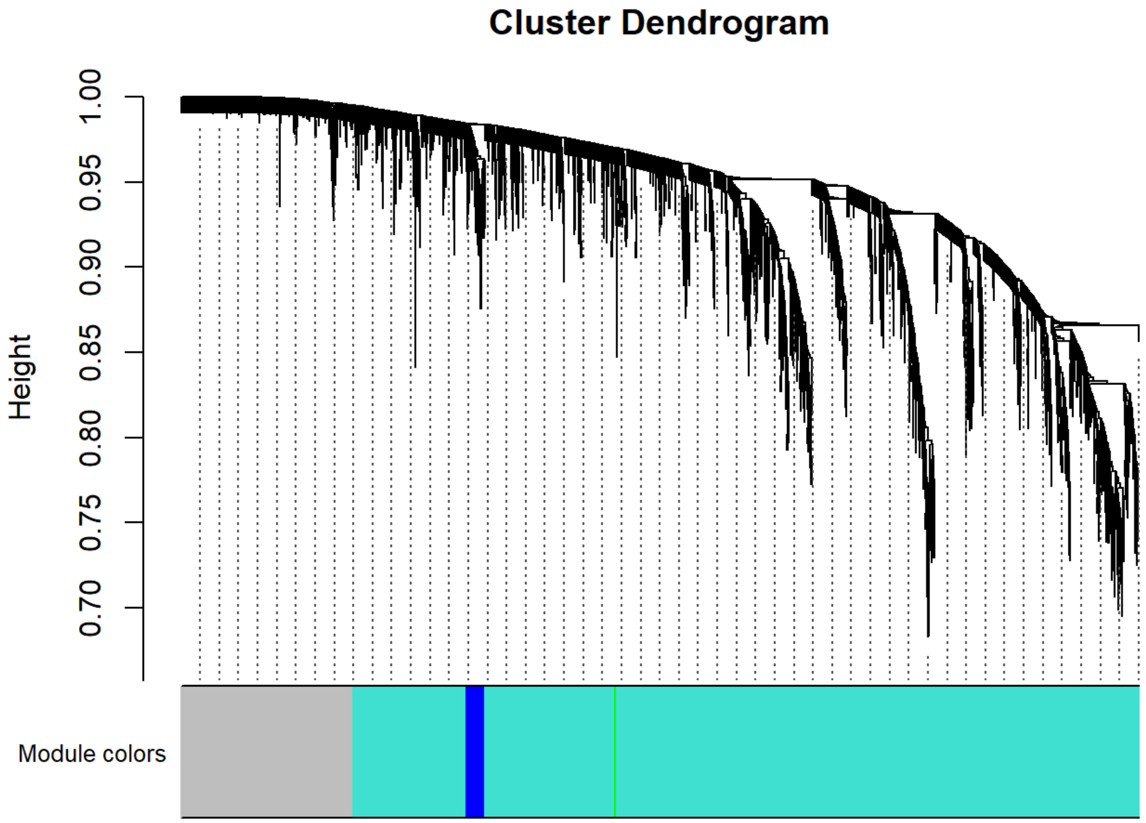

Supplementary Figure S2. Brain hippocampus.

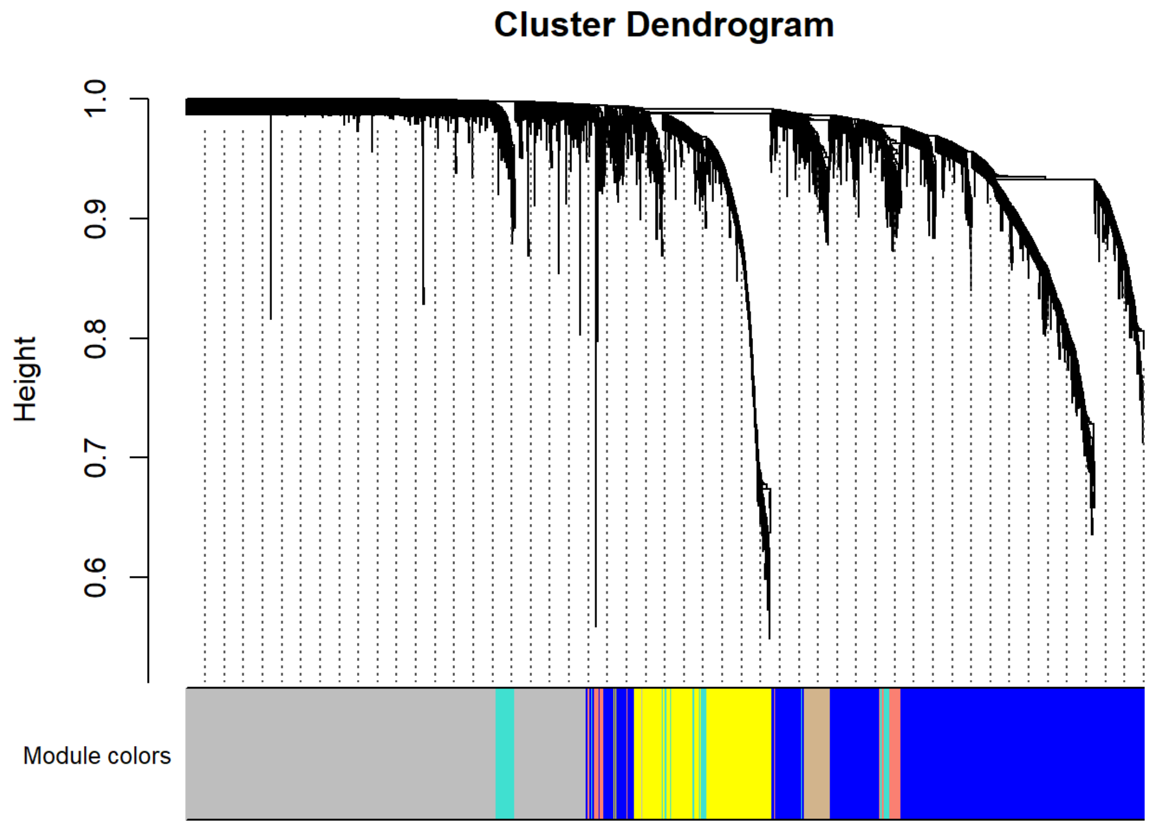

Supplementary Figure S3. Brain hypothalamus.

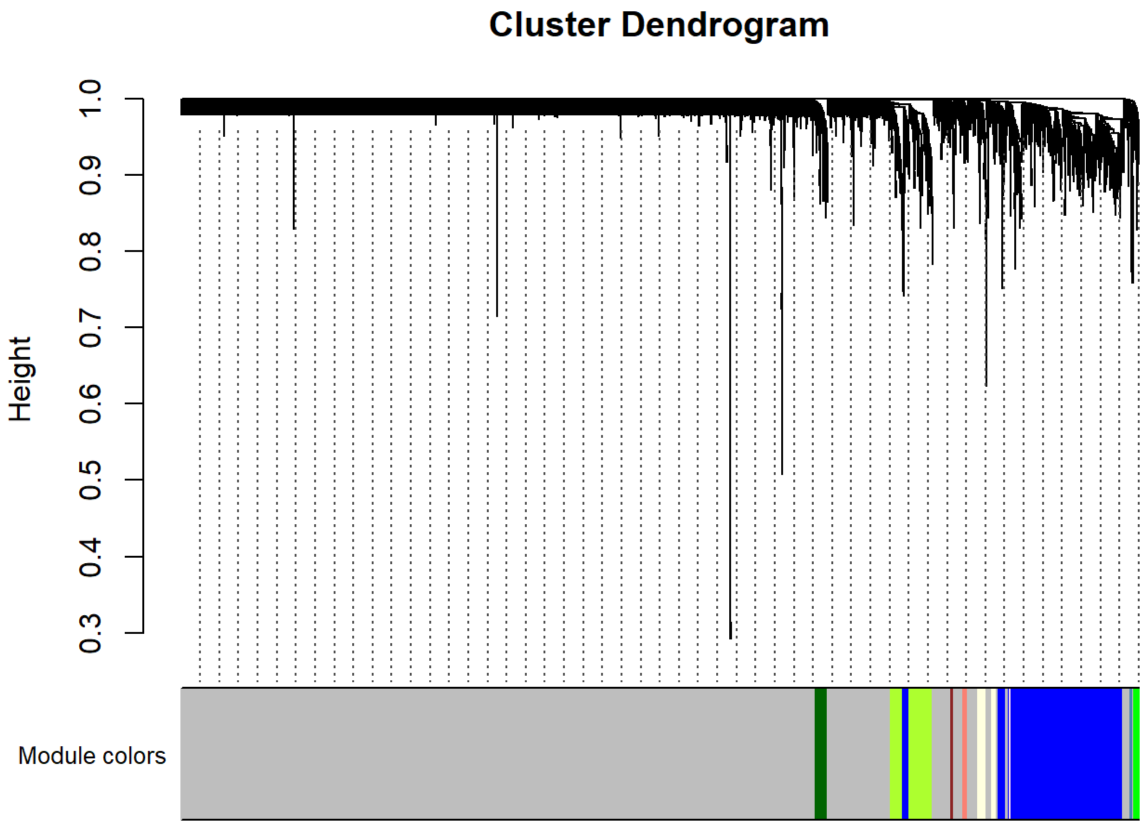

Supplementary Figure S4. Brain cortex.

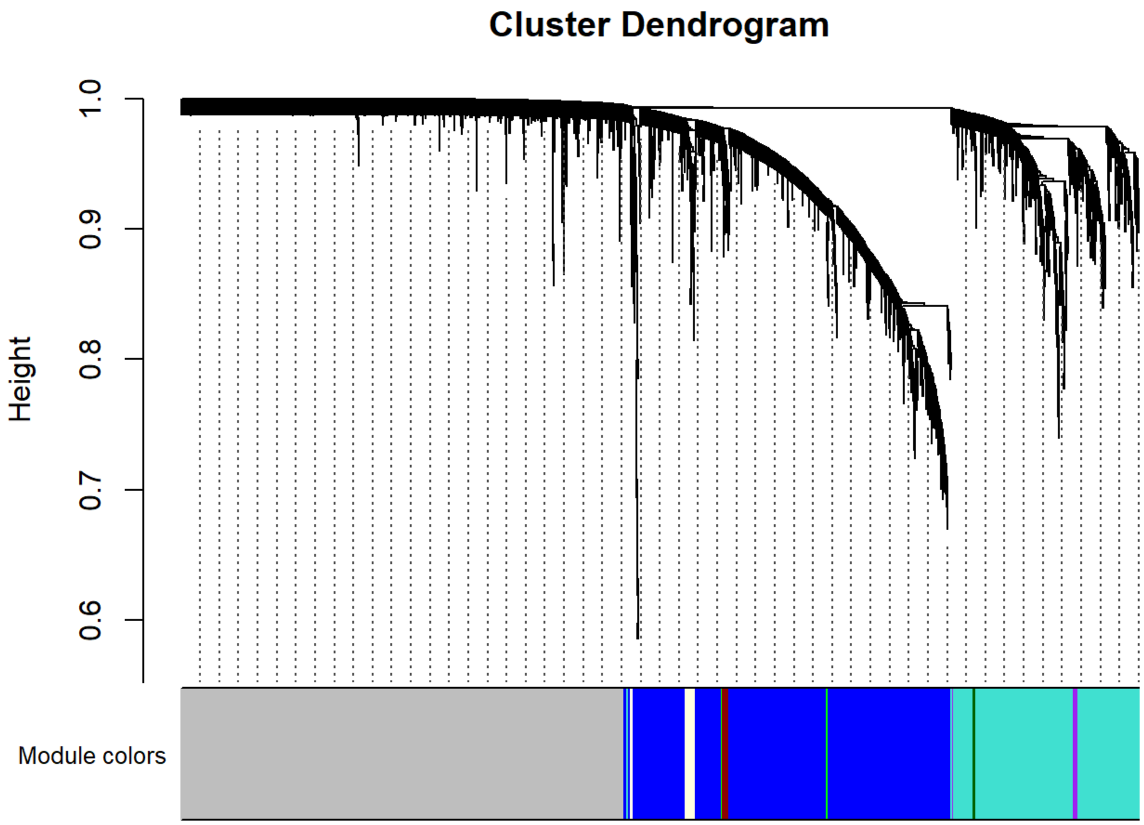

Supplementary Figure S5. Brain Frontal cortex.

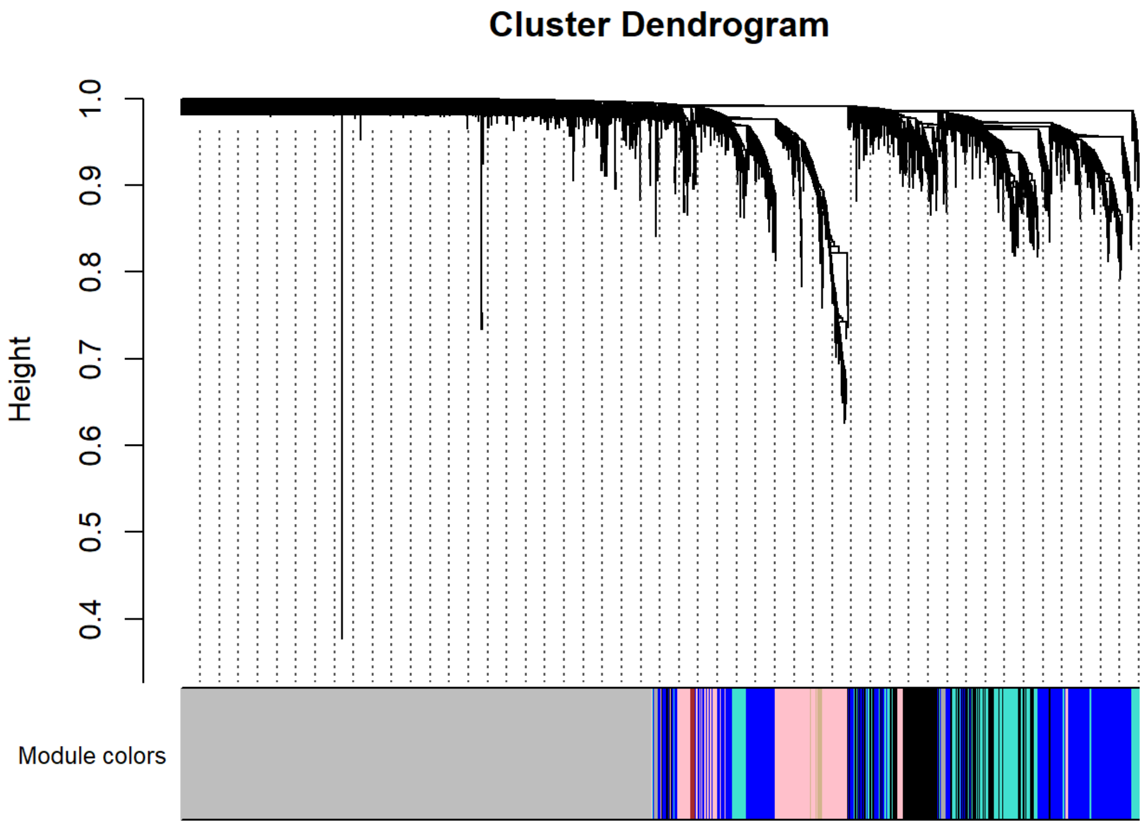

Supplementary Figure S6. Brain Anterior cingulate cortex.

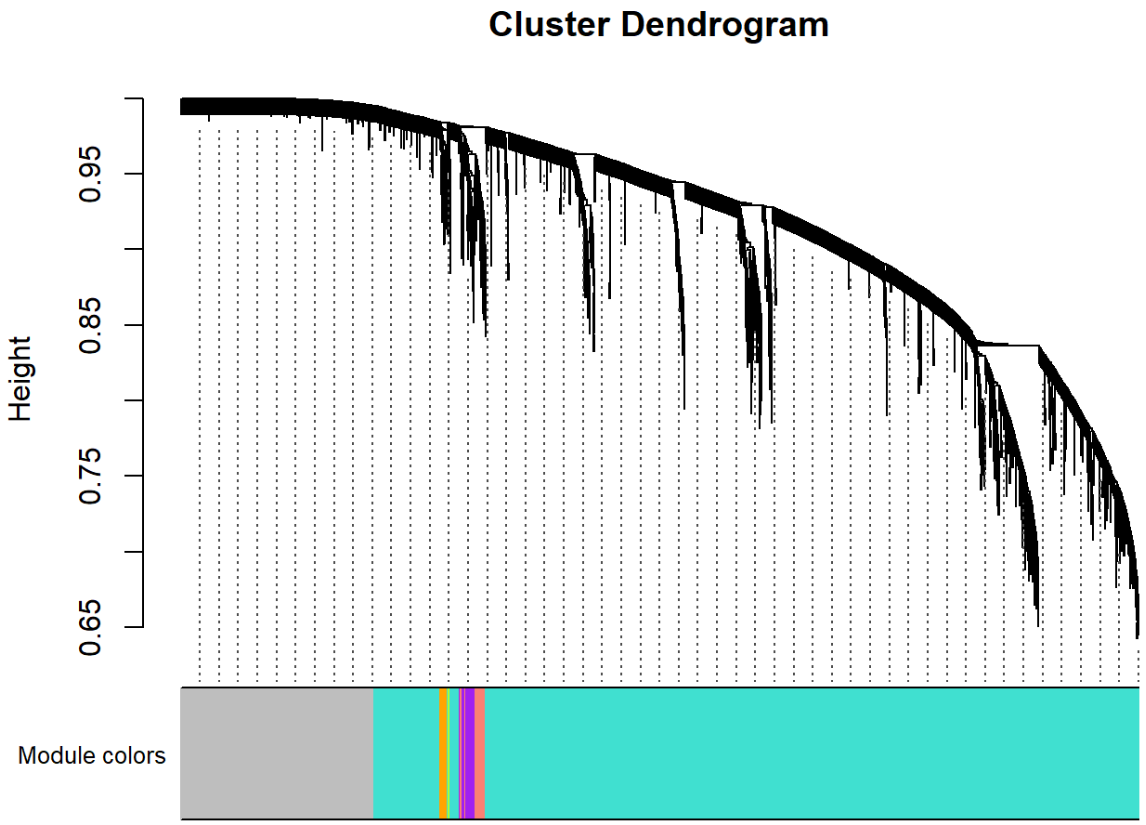

Supplementary Figure S7. Brain caudate.

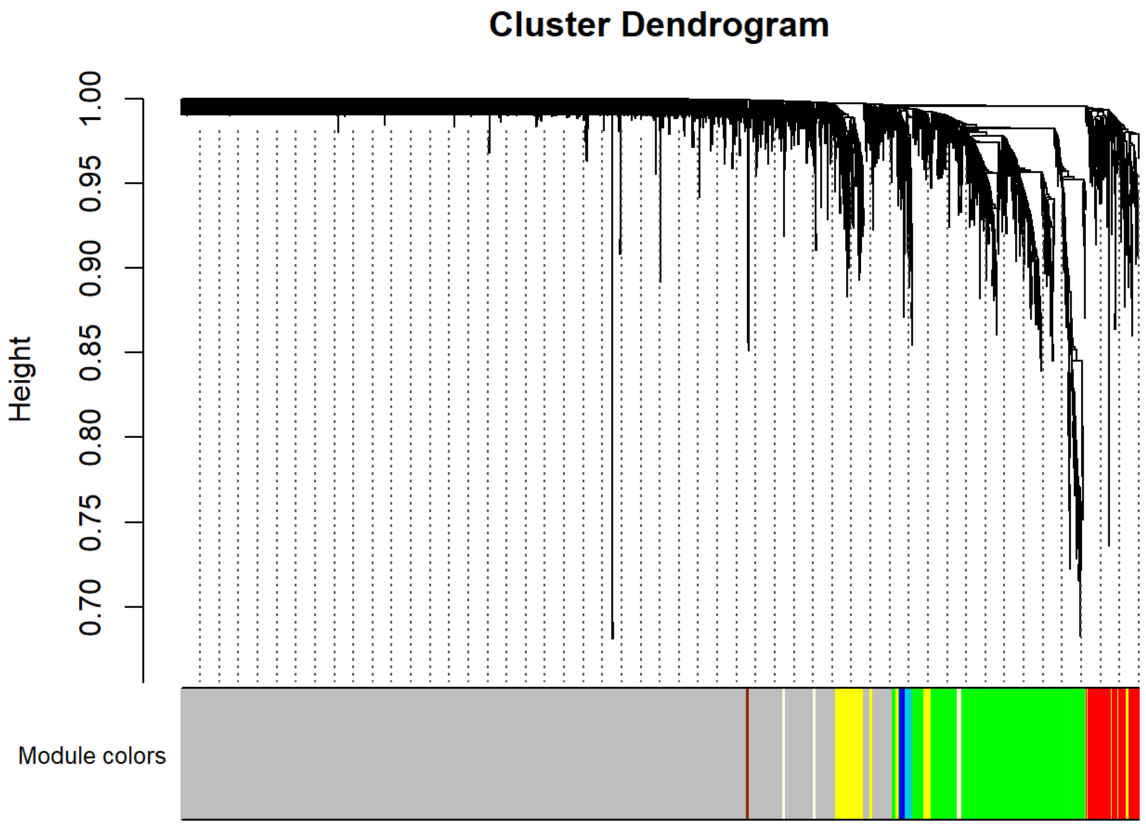

Supplementary Figure S8. Brain cerebellar hemisphere.

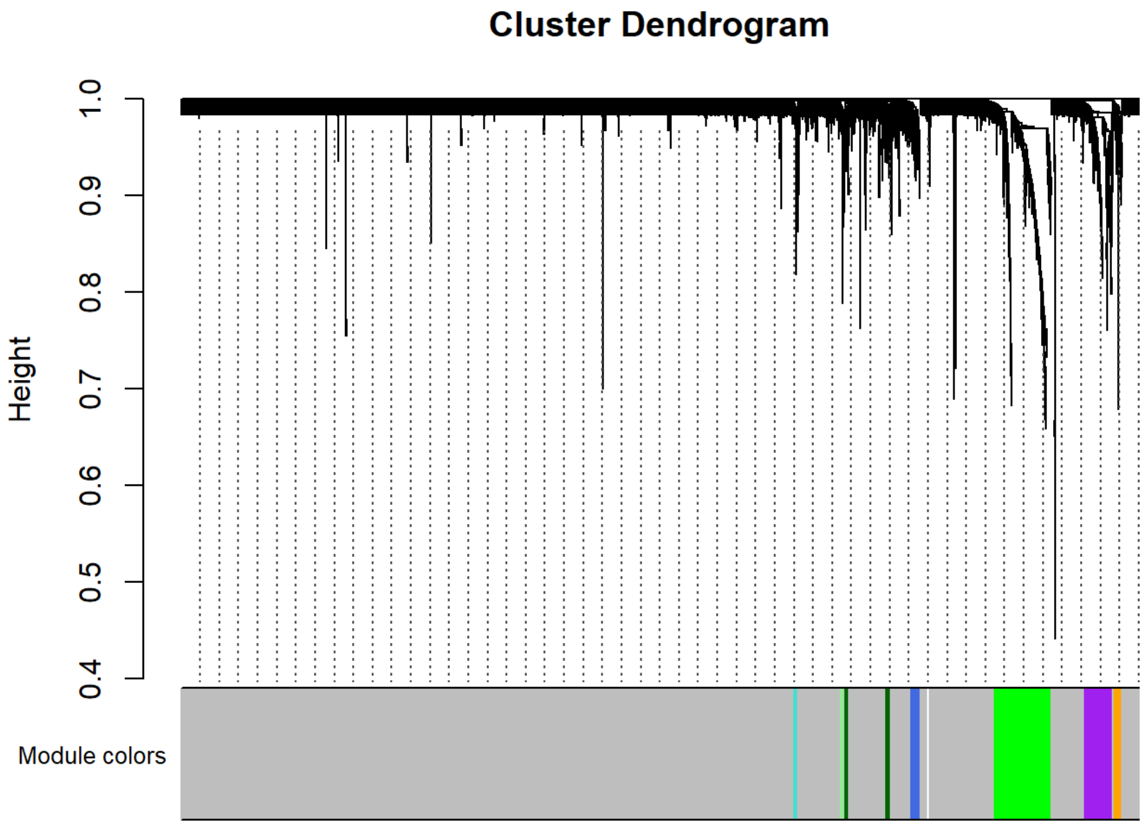

Supplementary Figure S9. Brain cerebellum.

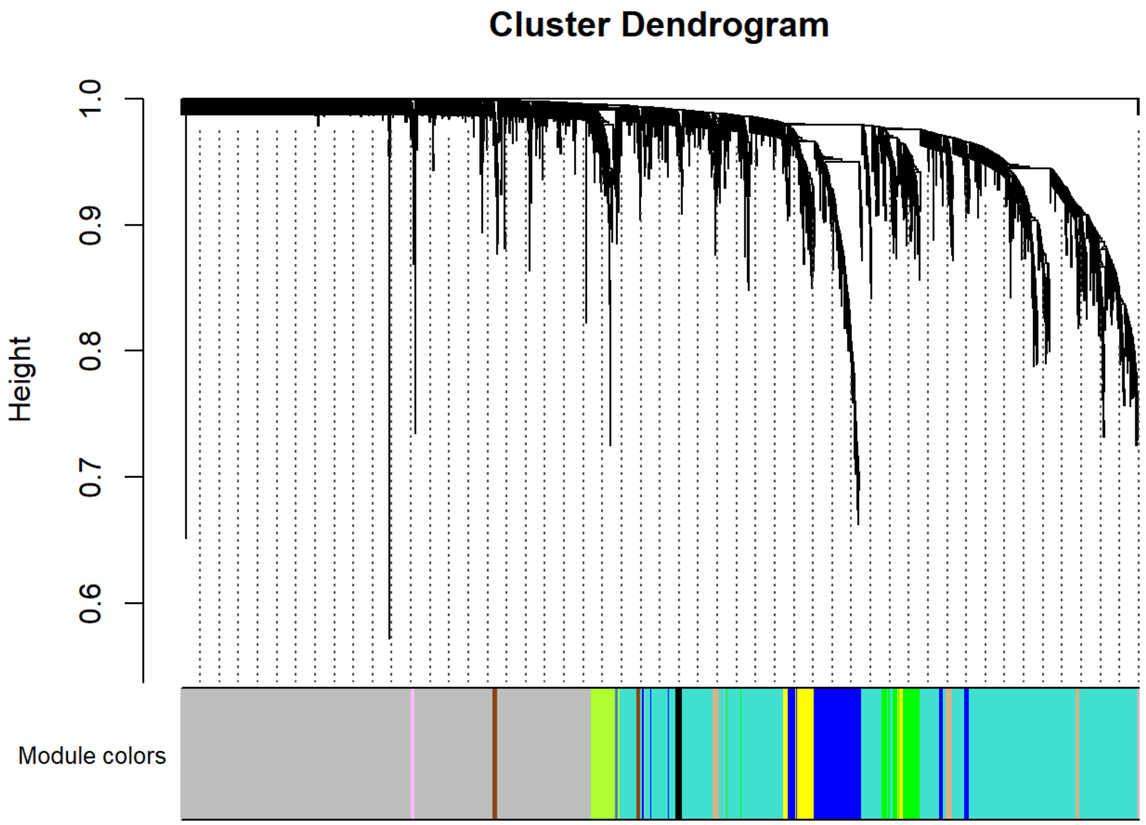

Supplementary Figure S10. Brain nucleus accumbens.

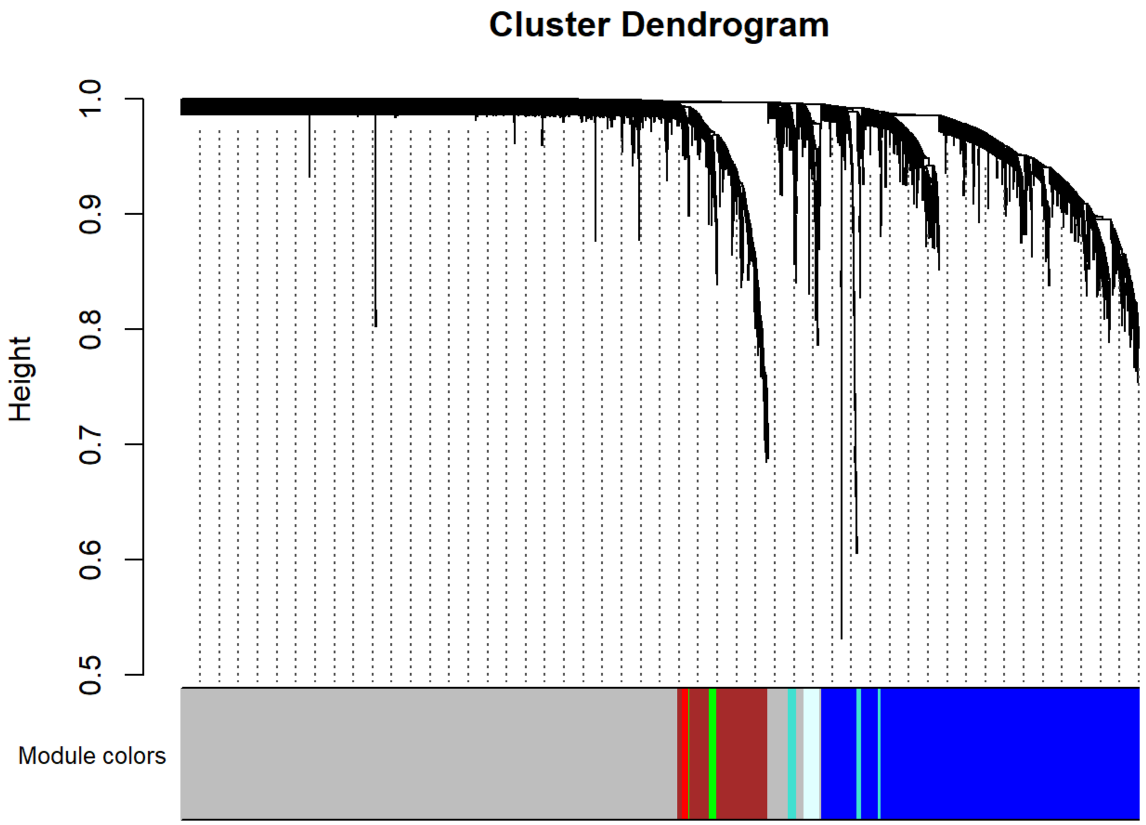

Supplementary Figure S11. Brain putamen.

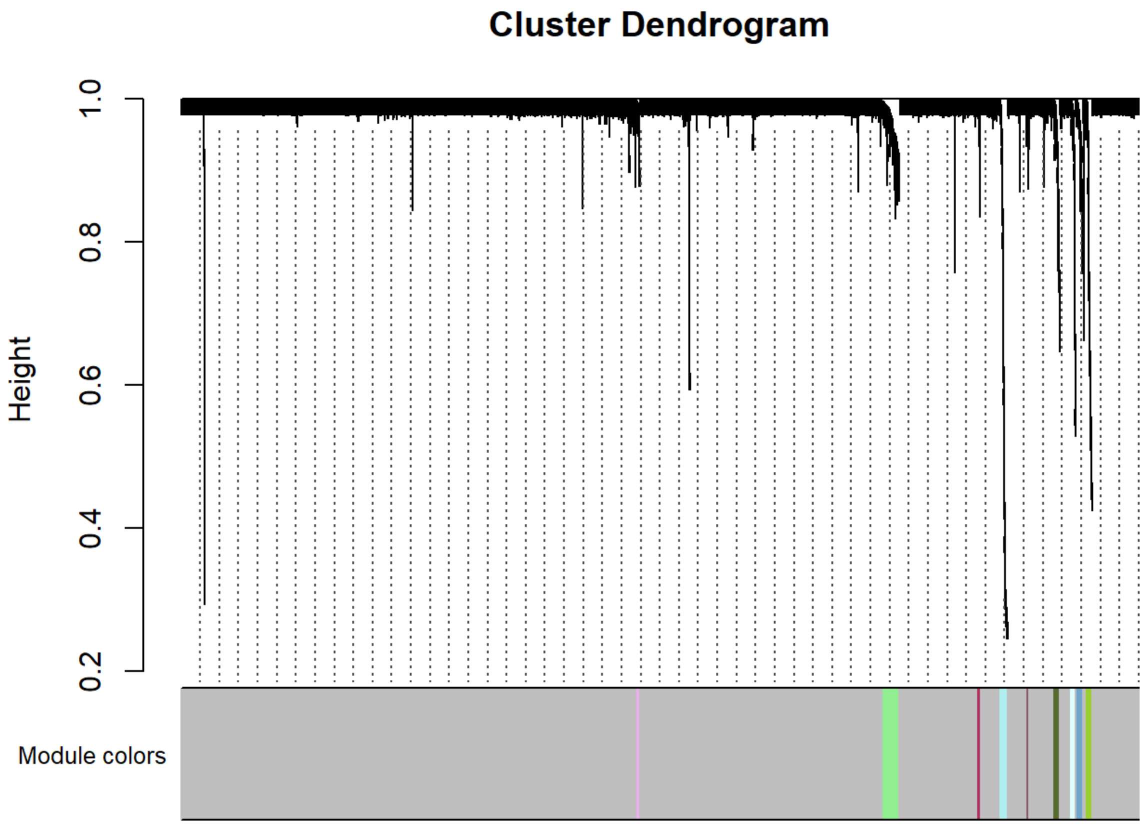

Supplementary Figure S12. Brain substantia nigra.

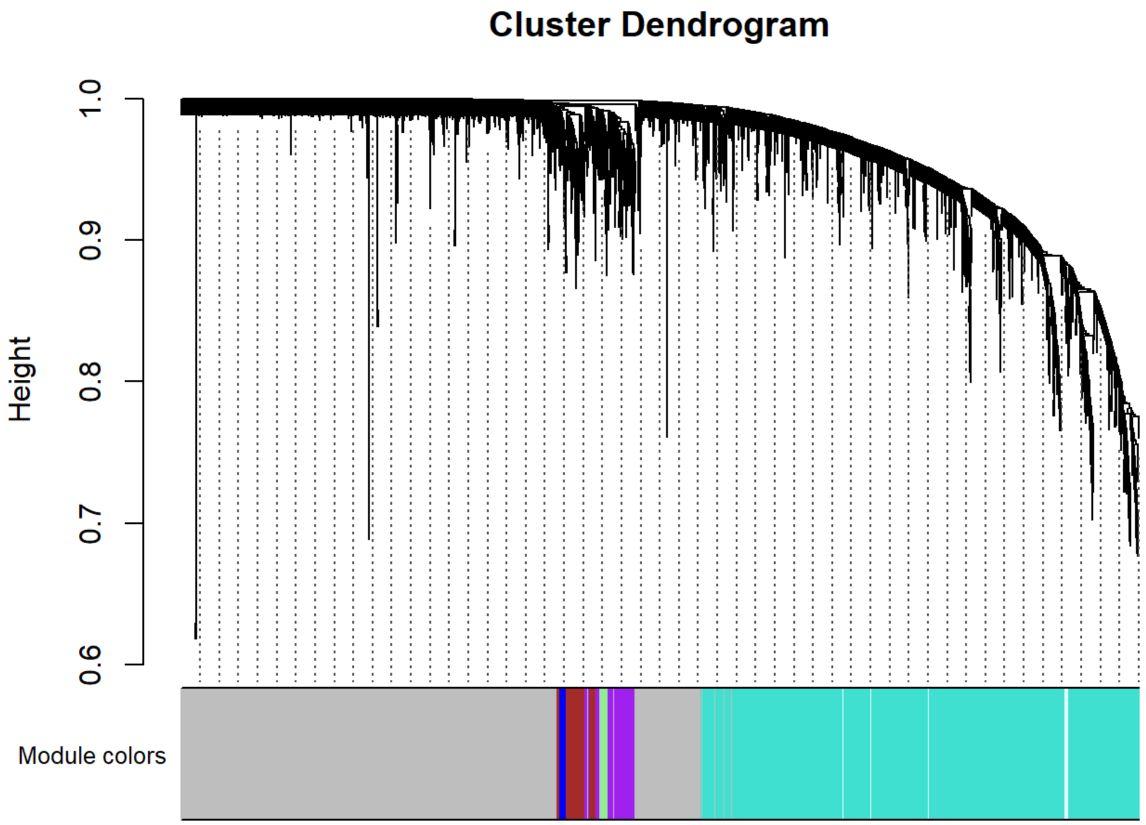

Supplementary Figure S13. Brain spinal cord.

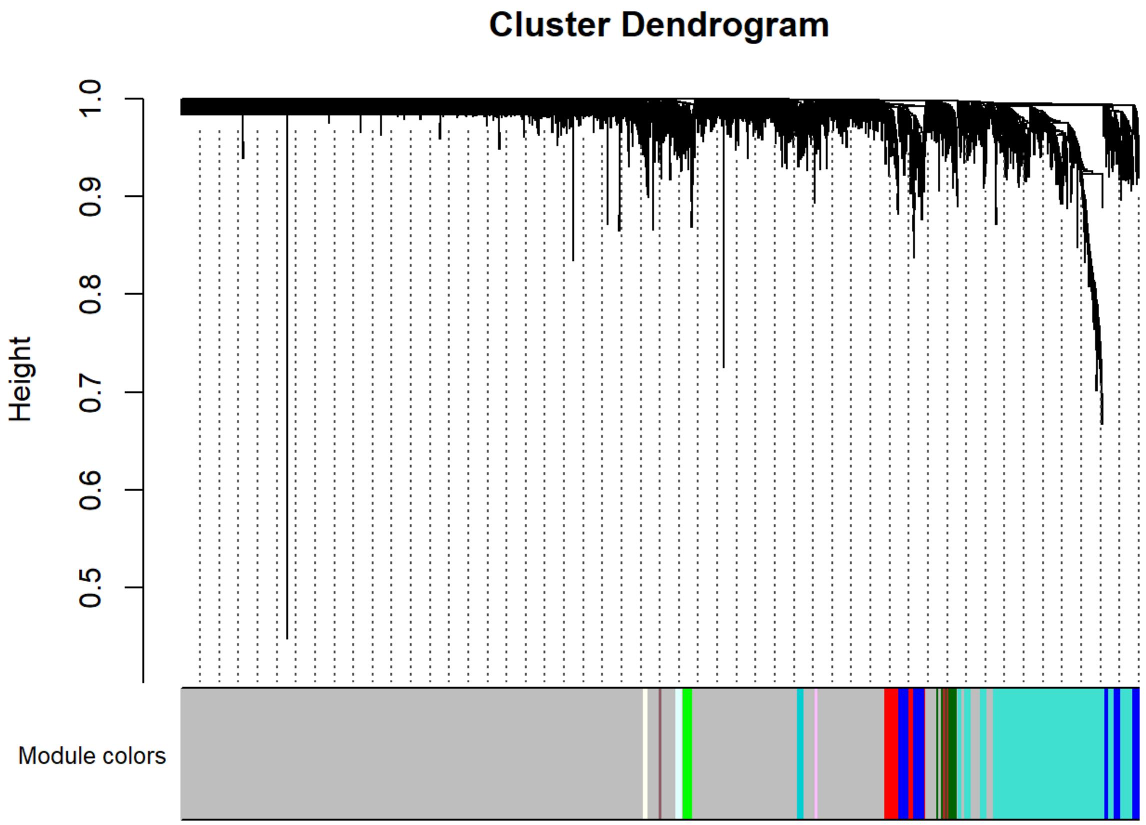

Supplementary Figure S14. Nerve tibial.

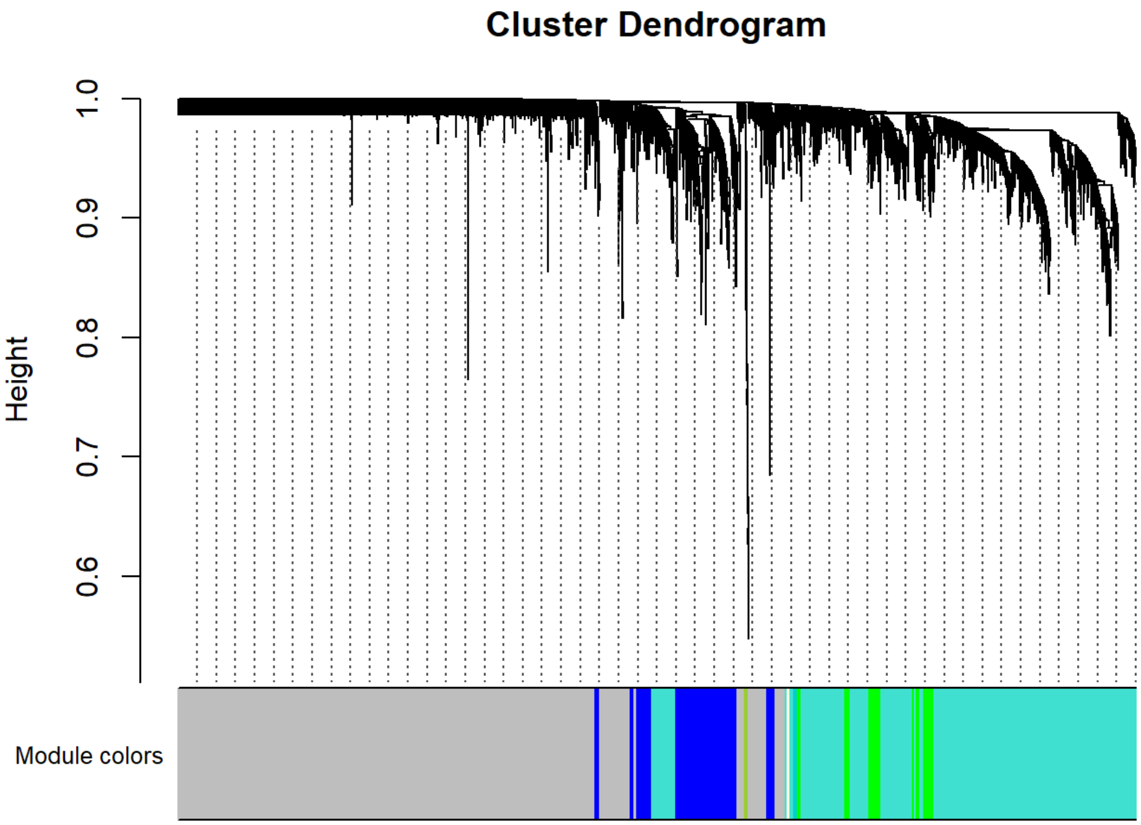

Supplementary Figure S15. Whole blood.
