# Supplementary material for: Shared Genetic Regulatory Networks Contribute to Neuropathic and Inflammatory Pain: Multi-Omics Systems Analysis
Source: Biomolecules. 2022 Oct 11;12(10):1454. doi: 10.3390/biom12101454 (PMC9599593; doi:10.3390/biom12101454)
Supplement: Supplementary file 1 [file biomolecules-12-01454-s001.zip › biomolecules-1926396-supplementaryS2.pdf]

Supplementary Material

We have verified the expression pattern of *C5ar1* and *Serpine1* using pain models in vitro. The mRNA expression level of *C5ar1* and *Serpine1* on the injured side was downregulated, which is, to a certain extent, consistent with the report of Parisien M et al.

Methods

SNI surgery was performed as previously described (PMID: 10924808). On postoperative day 2, rats were deeply anesthetized with 2.5% avertin (0.2ml /10g body weight) and were perfused transcardially with PBS (4℃,1 mL/g bodyweight). The spinal cord lumbar enlargement was harvested rapidly on ice. Total RNA was isolated from homogenized lumbar enlargement using Kimble Pellet Pestle Cordless Motor homogenizer (DWK Life Sciences) and extracted using RNeasy Plus Micro Kit (Qiagen). cDNA was reverse-transcribed using random hexamers from total RNA using SuperScript III First-Strand Synthesis System (Invitrogen) per the manufacturer's protocol. Quantitative RT-PCR was performed using the Sybrgreen method on the CFX Connect Real-Time PCR Detection System (Bio-Rad).

The primers used were as follows: β-actin: forward primer,TGTCACCAACTGGGACGATA; reverse primer, GGGGTGTTGAAGGTCTCAAA. C5AR1: forward primer,AGCATTGCTCCTCACCATTC; reverse primer, TCACCACTTTGAGCGTCTTG. SERPINE1: forward primer, GGCACAATCCAACAGAGACAATC; reverse primer, AGGCTTCTCATCCCACTCTCAA. Target gene expression levels were normalized to β-actin internal control, and their relative expression ratios were reported as 2^(-δδCt).

Results

In the SNI model of neuropathic pain, we found that *C5ar1 and Serpine1* mRNA was downregulated in the ipsilateral spinal cord of the injured side in rats 2 days after surgery **(Fig. S16)**.

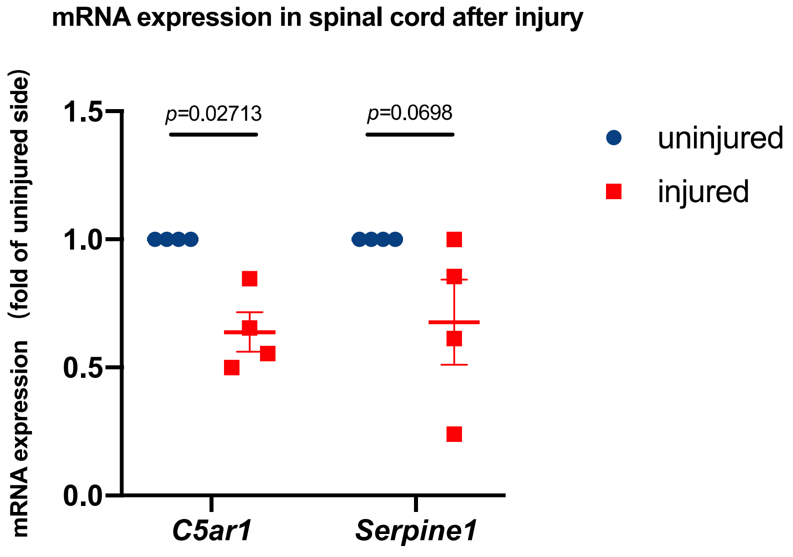

Figure S16. ***C5ar1*** **and** ***Serpine1*** **mRNA were downregulated in the ipsilateral spinal cord after SNI** qRT-PCR of ipsilateral and contralateral lumbar enlargement of *C5ar1 and Serpine1* mRNA in SD rats 2 days after injury. Data are shown as mean ± SEM. n=4 for each group. One-sided pared T-test analysis, p values are marked on top of the plots.
